# Altered placental expression of small humanin-like peptides in gestational diabetes mellitus

**DOI:** 10.48101/ujms.v131.14142

**Published:** 2026-07-03

**Authors:** Sude Dagli, Merve Ece Oyur, Bedircan Turan, Aysegul Turkkol, Umut Kerem Kolac

**Affiliations:** a Faculty of Medicine, Aydin Adnan Menderes University, Aydin, Türkiye; b Department of Medical Biology, Faculty of Medicine, Aydin Adnan Menderes University, Aydin, Türkiye; c Department of Biophysics, Faculty of Medicine, Aydin Adnan Menderes University, Aydin, Türkiye

**Keywords:** Gestational diabetes mellitus, placenta, small humanin-like peptides, insulin resistance

## Abstract

**Background:**

Gestational diabetes mellitus (GDM) is characterized by maternal insulin resistance and hyperglycemia and has been associated with placental mitochondrial alterations. Small humanin-like peptides (SHLP1–6), encoded within the mitochondrial 16S rRNA region, have been described as mitochondria-derived peptides (MDPs) with reported cytoprotective and metabolic effects in experimental settings. However, their expression in placental tissues from GDM pregnancies has not been previously investigated.

**Methods:**

Placental tissues were obtained from women with GDM and normoglycemic controls following cesarean delivery. Maternal fasting glucose, fasting insulin, glycated hemoglobin A1c (HbA1c), and the homeostatic model assessment for insulin resistance (HOMA-IR) were recorded. Total RNA was isolated from placental samples, and the mRNA expression levels of *SHLP1–6* were quantified using quantitative real-time polymerase chain reaction (PCR). In addition, Humanin and SHLP2 peptide levels were assessed at the protein level. Group comparisons and correlation analyses were performed to evaluate associations between placental *SHLP* expression and maternal metabolic parameters within each group.

**Results:**

Placental expression levels of all six *SHLP* transcripts were significantly reduced in the GDM group compared with controls. Lower placental *SHLP* expression was significantly associated with higher HOMA-IR and HbA1c values. In addition, reduced *SHLP* transcript levels were associated with increased neonatal birth weight. Consistently, Humanin and SHLP2 levels were significantly reduced in both serum and placental samples in the GDM group.

**Conclusions:**

This study demonstrates that placental mRNA levels of *SHLP*s are reduced in pregnancies complicated by GDM and are associated with maternal metabolic status. The integration of transcriptional findings with targeted peptide measurements supports the notion that altered placental SHLP expression is linked to metabolic disturbances in GDM and warrants further investigation into the role of MDPs in placental adaptation during diabetic pregnancy.

## Introduction

Diabetes associated with pregnancy represents a significant global health concern affecting millions of women worldwide. Diabetes during pregnancy can be defined either as pre-existing type 1 or type 2 diabetes (1). In addition, it may manifest as gestational diabetes mellitus (GDM), which develops in women without a prior history of diabetes and is typically diagnosed between the 26th and 28th weeks of gestation (2). The global prevalence of pregnancy-related diabetes has been rising rapidly, largely due to increasing rates of obesity (3). Pregnancy-related diabetes is associated with an increased risk of several chronic disorders in mothers later in life, including cardiovascular disease and metabolic syndrome (4). Insulin resistance associated with low-grade chronic inflammation during pregnancy is a hallmark of GDM and is known to impair placental endothelial cell function (3).

During pregnancy, the placenta serves as a critical interface between the mother and the fetus, playing an essential role in ensuring a successful gestational outcome (5). It facilitates the transfer of nutrients and oxygen from the mother to the fetus, removes fetal waste products, and protects the fetus from maternal inflammatory responses (6). Furthermore, the placenta senses metabolic alterations and secretes hormones that regulate maternal physiology to support optimal fetal development (7). Consequently, impairment of placental function in diabetic pregnancies may lead to complications such as pre-eclampsia, intrauterine growth restriction, and fetal macrosomia (8). Trophoblasts, among the most metabolically active cell types within the placenta, play vital roles in maternal–fetal metabolic regulation (9) and are essential for cholesterol and steroid-hormone synthesis and transport (10). Accordingly, these cells contain a highly developed network of mitochondria and endoplasmic reticulum (11).

Mitochondrial dysfunction has been implicated in the development of GDM, and accumulating evidence supports its contribution to the disease pathophysiology (12). Our group previously demonstrated that the expression levels of proteins involved in mitochondrial dynamics were significantly altered in placentas from women with GDM compared with non-diabetic controls (1). Mitochondria possess their own circular genome (mtDNA) of approximately 16.5 kilobases encoding 37 genes – 13 proteins of the respiratory chain, 22 tRNAs, and 2 rRNAs (13). Beyond their classical role in cellular energy production, several short open reading frames (sORFs) within mtDNA have been shown to encode mitochondria-derived peptides (MDPs), which have been implicated in cellular stress responses and metabolic regulation (14). To date, eight distinct MDPs have been characterized, with reported roles in cytoprotection and energy homeostasis (15). Among them, Humanin, a 24-amino-acid polypeptide encoded within the 16S rRNA region of mtDNA, has been associated with numerous homeostatic and cytoprotective functions, including promotion of cell survival (16), protection against oxidative stress, activation of chaperone-mediated autophagy (17), modulation of cellular redox balance, and inhibition of caspase-3 and -4 activation to prevent apoptosis (18).

Following the characterization of Humanin, further analysis of the mitochondrial *16S rRNA* region led to the identification of six additional Humanin-like peptides, collectively termed small Humanin-like peptides (SHLPs 1–6) (13). These bioactive peptides are encoded by sORFs (45–90 bp) embedded within the *12S rRNA* (generating mitochondrial open reading frame of the 12S rRNA-c [MOTS-c]) and *16S rRNA* (generating Humanin and SHLP1–6) genes. Translation of these peptides occurs in both mitochondrial and cytosolic compartments, and they have been detected in circulation as well as in various tissues (19). Functionally, SHLPs exhibit diverse cytoprotective properties, including reported associations with cellular stress responses (20, 21), insulin sensitivity (13), and metabolite profiles (22). Among these mitochondrial peptides, MOTS-c has attracted particular attention for its metabolic significance. A previous study reported that serum MOTS-c levels were significantly reduced in women with GDM during the first and second trimesters compared with non-GDM controls, and that peptide concentrations were positively correlated with insulin-sensitivity indices (23).

The present study aimed to investigate the transcriptional expression of mitochondria-derived *SHLP1–6* in placental tissues from women with GDM and healthy pregnancies. Specifically, we sought to determine whether alterations in *SHLP* expression are associated with key metabolic and clinical parameters, including insulin resistance, hemoglobin A1c (HbA1c) levels, and neonatal birth weight. By integrating quantitative gene expression analysis with protein-level measurements and correlation profiling, our findings indicate that placental SHLPs are altered in GDM, highlighting mitochondrial peptides as molecular features associated with placental metabolic adaptation in diabetic pregnancy.

## Materials and methods

### Patient characteristics and placental tissue collection

The study was approved by the Non-Interventional Clinical Research Ethics Committee of Aydın Adnan Menderes University (approval no: 2025/169; date: May 26, 2025) and conducted in accordance with the Declaration of Helsinki. All participants provided written informed consent prior to enrollment.

Pregnant women were recruited from the Department of Obstetrics and Gynecology at Aydın Adnan Menderes University Hospital during routine antenatal follow-up visits. All participants underwent a standard 75-g, 2-h oral glucose tolerance test (OGTT) at approximately the 28th week of gestation as part of routine clinical care.

Following OGTT screening, women diagnosed with GDM according to the American Diabetes Association (ADA) criteria were assigned to the GDM group, while women with normal glucose tolerance were included as controls. Study groups were formed after diagnosis, with consideration of baseline characteristics such as maternal age, body mass index (BMI), and early pregnancy metabolic parameters to minimize potential confounding effects.

Placental tissues were obtained from singleton pregnancies delivered by cesarean section at the same institution as previously described (1). The use of cesarean delivery allowed standardized tissue collection and minimized variability related to labor-associated physiological changes. Placental tissue samples were collected immediately after delivery from the central region of the fetal side, carefully cleared of decidual and amniotic membranes, rinsed in phosphate-buffered saline (PBS), and stored at −80°C until further analysis.

Demographic and obstetric characteristics included maternal age, gestational age at delivery, BMI across all trimesters, white blood cell (WBC) counts, and neonatal birth weight. Glycosylated HbA1c levels were measured during each trimester to assess maternal glycemic status.

### Inclusion and exclusion criteria

At approximately the 28th week of gestation, all participants underwent a standard 75-g, 2-h OGTT according to the diagnostic criteria of the ADA. The diagnosis of GDM was established when at least one of the following thresholds was met or exceeded: fasting glucose ≥ 92 mg/dL, 1-h ≥ 180 mg/dL, and 2-h ≥ 153 mg/dL. All women in the GDM group were managed with dietary intervention alone, and those requiring insulin therapy were excluded from the study. Additional exclusion criteria included autoimmune disease, cardiovascular disease, preeclampsia, malignancy, chronic illness, pre-existing type 1 or type 2 diabetes mellitus, and a prior history of cancer.

### Assessment of insulin resistance

Insulin resistance was evaluated during the third trimester using the homeostatic model assessment for insulin resistance (HOMA-IR) (23). Fasting venous blood samples were collected after an overnight fast within 48 h following GDM diagnosis and prior to the initiation of dietary treatment, ensuring that measurements reflected the untreated metabolic state. Serum insulin levels were determined using a human insulin enzyme-linked immunosorbent assay (ELISA) kit (Cat. No. E0010Hu, BT-LAB, China). Fasting plasma glucose concentrations were measured by standard enzymatic methods in the hospital’s central biochemistry laboratory. The HOMA-IR index was obtained by multiplying fasting insulin (µIU/mL) by fasting glucose (mg/dL) and dividing the product by 405. Higher HOMA-IR scores indicate greater insulin resistance.

### RNA isolation and quantitative real-time PCR analysis

Equal amounts of placental tissue were homogenized, and total RNA was extracted using RiboEx Trizol Reagent (GeneAll, Cat. No: 301-001, Republic of Korea) following the manufacturer’s instructions. RNA concentration and purity were determined spectrophotometrically. Complementary DNA (cDNA) was synthesized from the isolated RNA using the WizScript™ cDNA Synthesis Kit (Cat. No: W2211, Republic of Korea). The reverse transcription procedure consisted of incubation at 25°C for 10 min, followed by 37°C for 120 min. Quantitative real-time PCR (qPCR) was carried out with the WizPure™ qPCR Master Mix (SYBR Green, Cat. No: W1711R-5, Republic of Korea) on CFX96 Touch Real-Time PCR Detection System (Bio-Rad, USA). The amplification protocol included an initial denaturation at 95°C for 5 min, followed by 40 cycles of denaturation at 95°C for 30 s and combined annealing/extension at 56–60°C for 60 s. The specific primer pairs used for amplification of each target gene are listed in Supplementary Table 1. Cycle threshold (Ct) values were normalized to the geometric mean of *β-actin* (*ACTB*) and *glyceraldehyde-3-phosphate dehydrogenase (GAPDH)*, and relative mRNA expression levels were calculated using the 2^–ΔΔCt^ method. Each reaction was performed in triplicate.

### Assessment of peptide levels

Humanin and SHLP2 peptide levels were measured in third-trimester serum samples and placental tissue lysates using commercially available sandwich ELISA kits according to the manufacturers’ instructions. Humanin levels were determined using a Human putative Humanin peptide ELISA kit (Cat. No: ELK8996, ELK Biotech, China), and SHLP2 levels were measured using a human SHLP2 ELISA kit (Cat. No: MM-64757H2, Meimian, China).

For placental lysate preparation, tissue samples were rinsed in cold PBS, homogenized in PBS-based lysis buffer on ice, and centrifuged to remove cellular debris. The resulting supernatants were collected for ELISA analysis. Total protein concentrations in placental lysates were determined with Bradford assay separately, and peptide levels in tissue lysates were normalized to total protein content. Serum peptide concentrations were expressed as pg/mL. Optical density was measured at 450 nm, and peptide concentrations were calculated from standard curves generated for each assay.

### Statistical analysis

All statistical analyses were performed using GraphPad Prism version 9.0 (GraphPad Software, USA). The distribution of all data was first assessed for normality using the Shapiro–Wilk test (α = 0.05). Group comparisons were performed using an unpaired Student’s *t* test for normally distributed variables and the Mann–Whitney U test for variables that did not meet normality assumptions.

Associations between SHLP expression levels and clinical and metabolic parameters were evaluated separately for the GDM and control groups using Pearson’s correlation coefficient (*r*). For each analysis, the correlation coefficient (*r*), coefficient of determination (*R*^2^), 95% confidence intervals, and *P*-values were calculated and reported. These analyses were exploratory in nature and were performed independently within each group. Scatter plots with fitted linear regression lines were used to visualize the relationships.

All data were expressed as mean ± standard error of the mean (SEM) unless otherwise stated. A *P*-value of < 0.05 was considered statistically significant.

## Results

### Clinical and biochemical profile of the study groups

The demographic and metabolic characteristics of the participants are summarized in Table 1. The study included 20 healthy pregnant women and 19 patients with GDM diagnosed according to ADA criteria. No significant differences were observed between groups in maternal age, gestational week at delivery, BMI across trimesters, WBC counts, birth weight, amniotic fluid index, or Apgar scores (*P* > 0.05).

**Table 1:** Patient clinical characteristics.

Characteristics	Control	GDM
	(*n* = 20)	(*n* = 19)
Age (years)	30.85 ± 4.78	31.37 ± 4.21
1st Trimester BMI (kg/m^2^)	25.81 ± 2.03	26.53 ± 2.09
2nd Trimester BMI	26.35 ± 2.20	27.28 ± 1.45
3rd Trimester BMI	27.02 ± 1.68	28.27 ± 1.21
1st Trimester white blood cell (x10^9^/L)	9.13 ± 2.02	8.94 ± 1.71
2nd Trimester white blood cell	9.57 ± 1.65	9.62 ± 1.84
3rd Trimester white blood cell	10.00 ± 2.34	11.39 ± 2.44
1st Trimester HbA1c (%)	4.68 ± 0.43	4.94 ± 0.38
2nd Trimester HbA1c	4.94 ± 0.70	5.57 ± 0.48
3rd Trimester HbA1c	5.14 ± 0.49	6.55 ± 0.46*
3rd Trimester insulin (µIU/mL)	12.98 ± 0.64	17.41 ± 0.89*
Intrapartum amniotic fluid index (cm)	10.65 ± 2.64	11.68 ± 4.29
Gestational age (weeks)	37.80 ± 1.32	37.53 ± 0.96
Birth weight (g)	3070 ± 339.6	3188 ± 371.9
Apgar (1 min)	8.10 ± 0.85	7.73 ± 0.87
Apgar (5 min)	9.00 ± 0.79	8.84 ± 0.76
75 g OGTT (~ 28. weeks of gestation)		
Fasting (mg/dL)	80.65 ± 7.94	102.7 ± 10.85*
1 h	124.3 ± 10.54	191.7 ± 10.72*
2 h	103.5 ± 7.45	169.7 ± 13.45*

Data are presented as mean ± SD. Significant differences were found *P < 0.05 compared with control. Control: normal pregnant women; GDM: gestational diabetes mellitus; BMI: body mass index; HbA1c: glycated hemoglobin; OGTT: oral glucose tolerance test; Apgar: (appearance, pulse grimace response, activity, respiration) newborn’s status after birth. Statistical analysis was performed using unpaired Student’s *t* test.

**Figure 1:**
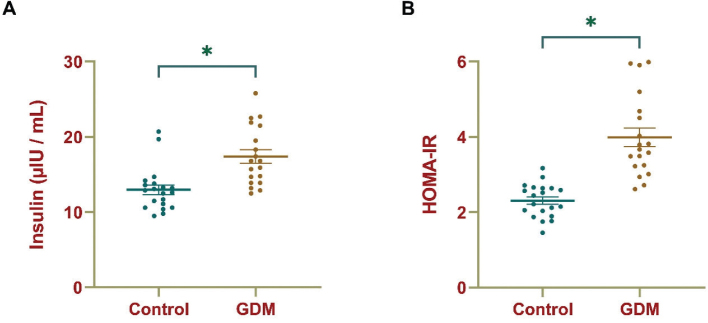
Assessment of insulin resistance in control and GDM pregnancies. (A) Serum insulin concentrations and (B) HOMA-IR values in control (n = 20) and GDM (n = 19) groups during the third trimester. Fasting venous blood samples were obtained after overnight fasting. Serum insulin levels were quantified using a human insulin ELISA kit, and fasting plasma glucose levels were measured. The HOMA-IR index was calculated as [fasting insulin (µIU/mL) × fasting glucose (mg/dL)] / 405, with higher scores indicating increased insulin resistance. Data are presented as mean ± SEM. *P < 0.05 compared to control group (unpaired two-tailed t test). GDM: Gestational diabetes mellitus; HOMA-IR: homeostatic model assessment for insulin resistance; SEM: standard error of the mean.

Metabolic evaluation revealed that women with GDM exhibited a clear pattern of glucose dysregulation and insulin resistance. Specifically, third-trimester HbA1c levels were significantly elevated in the GDM group compared with controls (6.55 ± 0.46% vs. 5.14 ± 0.49%, *P* < 0.05), while fasting, 1-h, and 2-h plasma glucose levels during the 75-g OGTT were markedly higher (*P* < 0.05 for all). Consistent with these findings, third-trimester serum insulin concentrations and HOMA-IR values were significantly higher in the GDM group compared with controls (insulin: 17.41 ± 0.89 vs. 12.98 ± 0.64 µIU/mL; HOMA-IR: 3.99 ± 0.24 vs. 2.30 ± 0.09; *P* < 0.05 for both), indicating the presence of both hyperinsulinemia and hyperglycemia (Table 1 and Figure 1A–B).

In contrast, third-trimester aspartate aminotransferase (AST) and alanine aminotransferase (ALT) activities did not differ significantly between the groups, suggesting preserved hepatic function (Supplementary Figure 1A–B).

Taken together, these data confirm that the GDM cohort displayed the classical biochemical phenotype of GDM – characterized by hyperglycemia and elevated insulin resistance – without evidence of hepatic dysfunction that could confound the interpretation of placental mitochondrial gene expression analyses.

### Downregulation of placental SHLP transcripts in GDM and its relationship with peptide levels and mitochondrial transcript context

**Figure 2:**
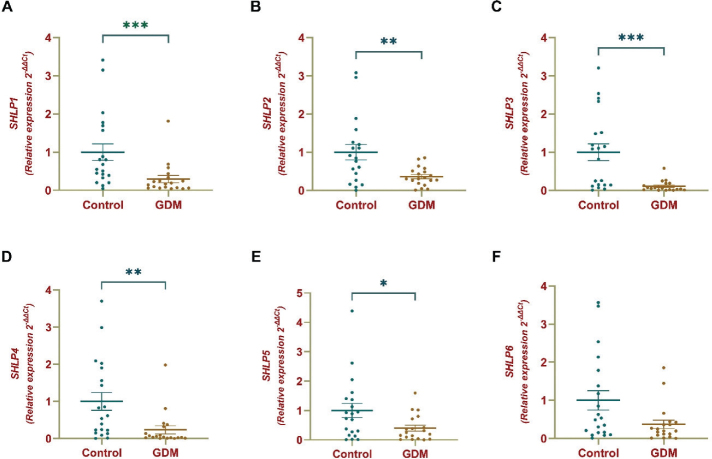
Placental SHLP1–6 transcript levels in control and GDM groups. Relative mRNA expression levels of (A) *SHLP1*, (B) *SHLP2*, (C) *SHLP3*, (D) *SHLP4*, (E) *SHLP5*, and (F) *SHLP6* in placental tissues from control (*n* = 20) and GDM (*n* = 19) pregnancies. Gene expression levels were normalized to the geometric mean of *ACTB* and *GAPDH* and calculated using the 2^– ΔΔCt^ method. Expression values are presented relative to the mean expression of the control group, which was set to 1. Each reaction was performed in triplicate. Data are presented as mean ± SEM. *P < 0.05, **P < 0.01, ***P < 0.001 vs control (Mann–Whitney U test). SHLP: Small humanin-like peptides; GDM: Gestational diabetes mellitus; SEM: standard error of the mean.

Placental expression levels of mitochondria-derived *SHLP1–6* transcripts were first evaluated to determine whether GDM is associated with alterations in mtDNA-encoded peptide transcription (Figure 2). qPCR analysis revealed a consistent and significant downregulation of SHLP transcripts in placental tissues from women with GDM compared with controls. Specifically, *SHLP1, SHLP2, SHLP3, SHLP4, SHLP5*, and *SHLP6* transcript levels were reduced by approximately 71, 64, 90, 77, 61, and 63%, respectively. Among these, *SHLP1* and *SHLP3* exhibited the most pronounced decreases (*P* < 0.001), whereas *SHLP2* and *SHLP4* were also significantly reduced (*P* < 0.01). *SHLP5* showed a moderate but significant decrease (*P* < 0.05), while *SHLP6* displayed a similar downward trend that did not reach statistical significance. These findings indicate a robust and coordinated suppression of *SHLP* transcript levels in GDM placentas.

**Figure 3:**
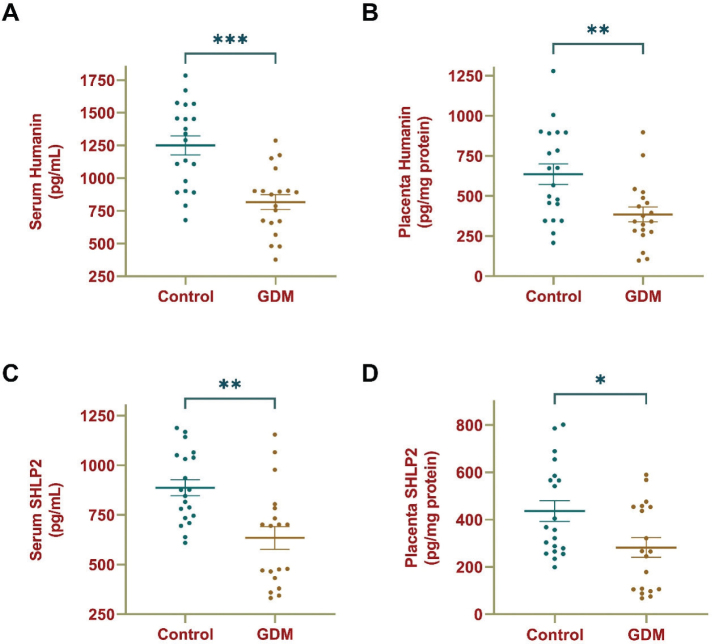
Serum and placental Humanin and SHLP2 peptide levels in control and GDM groups. Peptide levels of (A) serum Humanin, (B) placental Humanin, (C) serum SHLP2, and (D) placental SHLP2 in control (*n* = 20) and GDM (*n* = 19) pregnancies were measured by ELISA. Serum peptide concentrations are presented as pg/mL, whereas placental peptide levels were normalized to total protein content and expressed as pg/mg protein. Each sample was measured in duplicate. Data are presented as mean ± SEM. *P < 0.05, **P < 0.01, ***P < 0.001 vs control (Mann–Whitney U test). SHLP: Small humanin-like peptides; GDM: Gestational diabetes mellitus; SEM: standard error of the mean.

To determine whether the observed transcriptional changes are reflected at the peptide level, circulating and placental concentrations of Humanin and SHLP2 were measured by ELISA (Figure 3). Both serum and placental levels of Humanin were significantly decreased in the GDM group compared with controls (*P* < 0.001 and *P* < 0.01, respectively). Similarly, SHLP2 concentrations were significantly reduced in both serum and placental lysates in GDM pregnancies (*P* < 0.01 and *P* < 0.05, respectively). These findings demonstrate that the transcriptional downregulation of SHLPs is accompanied by a corresponding decrease at the peptide level, supporting the biological relevance of these changes.

**Figure 4:**
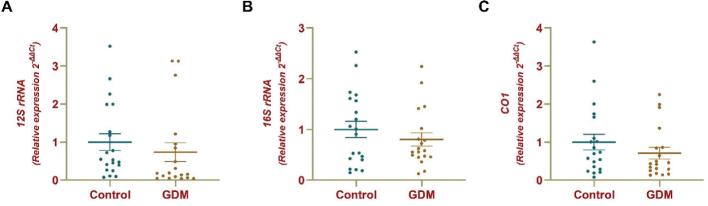
Placental mitochondrial transcript levels in control and GDM groups. Relative mRNA expression levels of (A) *12S rRNA*, (B) *16S rRNA* using primers targeting a *non-SHLP* region of *MT-RNR2*, and (C) *CO1* in placental tissues from control (n = 20) and GDM (n = 19) pregnancies. Gene expression levels were normalized to the geometric mean of *ACTB* and *GAPDH* and calculated using the 2^– ΔΔCt^ method. Expression values are presented relative to the mean expression of the control group, which was set to 1. Each reaction was performed in triplicate. Data are presented as mean ± SEM. GDM: Gestational diabetes mellitus; SEM: standard error of the mean.

To assess whether the reduction in *SHLP* transcripts reflects a global decrease in mitochondrial transcription or a more specific regulatory effect, the expression levels of key mitochondrial transcripts, including *12S rRNA, 16S rRNA*, and *cytochrome c oxidase I (CO1)*, were analyzed (Figure 4). Compared with controls, *12S rRNA, 16S rRNA*, and *CO1* expression levels were reduced by approximately 27, 20, and 29%, respectively; however, none of these changes reached statistical significance. This suggests that the marked reduction in *SHLP* expression cannot be fully explained by a generalized decrease in mitochondrial RNA abundance.

To further control for mitochondrial transcriptional variability, *SHLP* transcript levels were additionally normalized to mitochondrial transcripts (*16S rRNA* and *CO1*) (Supplementary Figure 2). When normalized to the non-SHLP region of *16S rRNA, SHLP1–6* expression levels were reduced by approximately 75, 64, 92, 82, 67, and 72%, respectively, with statistically significant decreases observed for *SHLP1–4*. Similarly, normalization to *CO1* revealed reductions of approximately 75, 71, 89, and 69% for *SHLP1–4*, whereas *SHLP5* and *SHLP6* showed only minor decreases (26 and 24%, respectively) and did not reach statistical significance. These findings indicate that the observed downregulation of *SHLP* transcripts in GDM is largely preserved even after accounting for mitochondrial transcript levels, supporting the notion of a more specific regulatory mechanism affecting mtDNA-encoded peptide expression.

### Correlation analysis between SHLP expression and metabolic and clinical parameters

**Figure 5:**
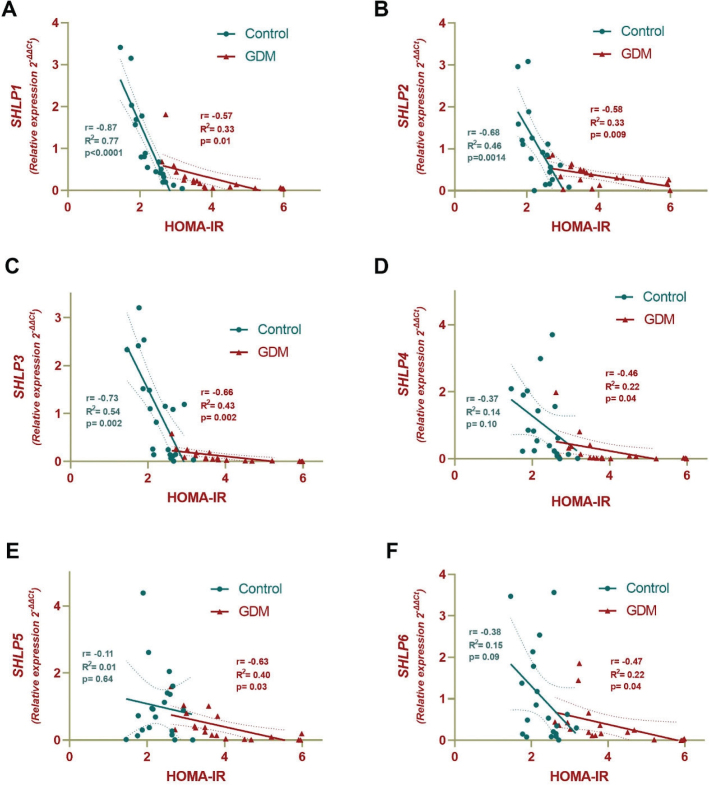
Correlations between placental *SHLP1–6* transcript levels and HOMA-IR index in control and GDM groups. Correlation analysis between placental mRNA expression of (A) *SHLP1*, (B) *SHLP2*, (C) *SHLP3*, (D) *SHLP4*, (E) *SHLP5*, and (F) *SHLP6* and the HOMA-IR index in control and GDM pregnancies. *SHLP* transcript levels were quantified by qPCR, normalized to the geometric mean of *ACTB* and *GAPDH*, and expressed as relative values calculated using the 2^– ΔΔCt^ method, with the control group used as the calibrator. Each data point represents an individual placenta (control, *n* = 20; GDM, *n* = 19). Pearson correlation analysis was performed to assess associations between variables within each group. Linear regression lines are shown for visualization purposes. The correlation coefficient (*r*), coefficient of determination (*R*^2^), and *P*-values were calculated using Pearson’s correlation test. Dotted lines represent 95% confidence intervals. SHLP: Small humanin-like peptides; GDM: Gestational diabetes mellitus; HOMA-IR: homeostatic model assessment for insulin resistance; qPCR: Quantitative real-time PCR.

Correlation analyses were performed separately within each group to explore the relationship between placental *SHLP* transcript levels and metabolic parameters. Consistent inverse associations were observed between SHLP expression and insulin resistance indices (Figure 5). In particular, *SHLP1, SHLP2*, and *SHLP3* exhibited strong and statistically significant negative correlations with HOMA-IR values in both control and GDM groups, indicating that higher insulin resistance is associated with lower placental *SHLP* transcript levels (Figure 5A–C). *SHLP4* also demonstrated a significant inverse correlation with HOMA-IR in the GDM group, whereas this association did not reach statistical significance in controls (Figure 5D). Similarly, *SHLP5* and *SHLP6* showed moderate but significant negative correlations with HOMA-IR in the GDM group, while these associations were weaker and non-significant in controls (Figure 5E, F).

**Figure 6:**
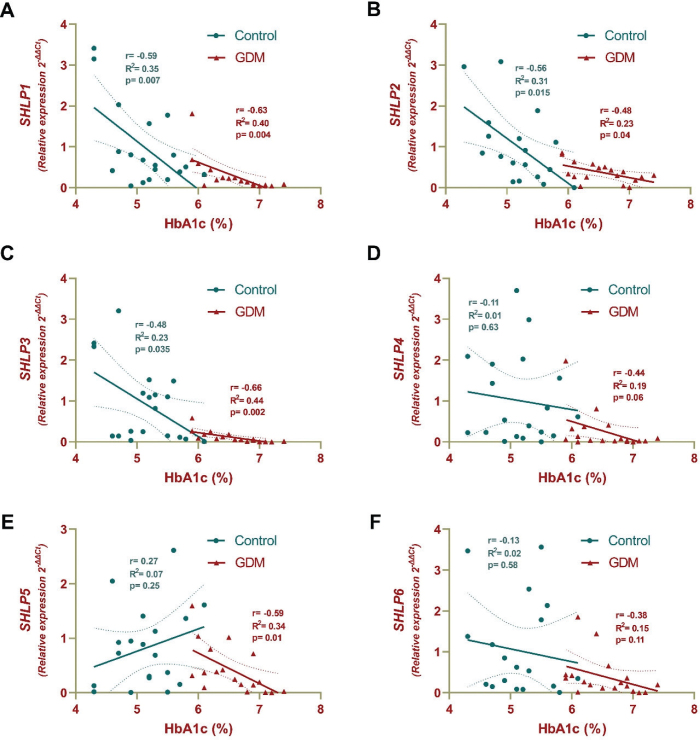
Correlations between placental *SHLP1–6* transcript levels and third-trimester HbA1c in control and GDM groups. Correlation analysis between placental mRNA expression of (A) *SHLP1*, (B) *SHLP2*, (C) *SHLP3*, (D) *SHLP4*, (E) *SHLP5*, and (F) *SHLP6* and maternal third-trimester HbA1c levels in control and GDM pregnancies. *SHLP* transcript levels were quantified by qPCR, normalized to the geometric mean of *ACTB* and *GAPDH*, and expressed as relative values calculated using the 2^– ΔΔCt^ method, with the control group used as the calibrator. Each data point represents an individual placenta (control, *n* = 20; GDM, *n* = 19). Pearson correlation analysis was performed to assess associations between variables within each group. Linear regression lines are shown for visualization purposes. The correlation coefficient (*r*), coefficient of determination (*R*^2^), and *P*-values were calculated using Pearson’s correlation test. Dotted lines represent 95% confidence intervals. SHLP: Small humanin-like peptides; GDM: Gestational diabetes mellitus; qPCR: Quantitative real-time PCR; HbA1c: hemoglobin.

As illustrated in Figure 6, within-group analyses revealed that *SHLP1–3* transcript levels were significantly inversely correlated with third-trimester HbA1c levels in both control and GDM groups. These findings indicate that increasing maternal glycemic burden is associated with reduced placental *SHLP* expression (Figure 6A–C). *SHLP5* displayed a moderate but significant negative correlation with HbA1c in the GDM group, whereas *SHLP4* and *SHLP6* did not show statistically significant associations (Figure 6D–F).

**Figure 7:**
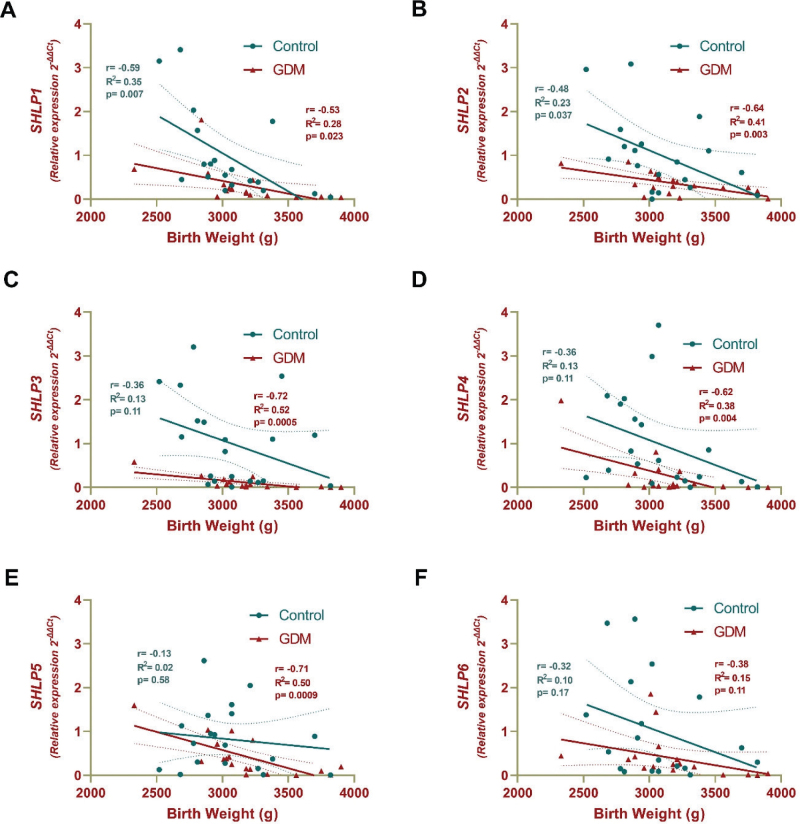
Correlations between placental *SHLP1–6* transcript levels and neonatal birth weight in control and GDM groups. Correlation analysis between placental mRNA expression of (A) *SHLP1*, (B) *SHLP2*, (C) *SHLP3*, (D) *SHLP4*, (E) *SHLP5*, and (F) *SHLP6* and neonatal birth weight measured at delivery in control and GDM pregnancies. *SHLP* transcript levels were quantified by qPCR, normalized to the geometric mean of *ACTB* and *GAPDH*, and expressed as relative values calculated using the 2^– ΔΔCt^ method, with the control group used as the calibrator. Each data point represents an individual placenta (control, *n* = 20; GDM, *n* = 19). Pearson correlation analysis was performed to assess associations between variables within each group. Linear regression lines are shown for visualization purposes. The correlation coefficient (*r*), coefficient of determination (*R*^2^), and *P*-values were calculated using Pearson’s correlation test. Dotted lines represent 95% confidence intervals. SHLP: Small humanin-like peptides; GDM: Gestational diabetes mellitus; qPCR: Quantitative real-time PCR.

Placental *SHLP* transcript levels also demonstrated significant relationships with neonatal birth weight (Figure 7). *SHLP1–3* showed strong inverse correlations with birth weight in both groups, with particularly pronounced associations observed in the GDM group (Figure 7A–C). In addition, *SHLP4* and *SHLP5* exhibited significant negative correlations with birth weight in GDM pregnancies, while these associations were weaker or non-significant in controls (Figure 7D–E). *SHLP6* showed a modest negative trend that did not reach statistical significance (Figure 7F). These findings suggest that reduced placental *SHLP* expression is associated with increased fetal growth, particularly in the context of GDM.

Given that maternal leukocyte counts are commonly used as an indicator of low-grade systemic inflammation in metabolic disorders, correlation analyses were also performed between *SHLP* transcript levels and maternal third-trimester WBC counts (Supplementary Figure 3). In the control group, *SHLP1, SHLP3, SHLP4*, and *SHLP6* exhibited statistically significant positive correlations with WBC levels (*P* < 0.05), indicating that higher leukocyte counts were associated with increased placental *SHLP* levels under physiological conditions. *SHLP2* showed a similar positive trend that did not reach statistical significance, whereas SHLP5 did not show a statistically significant association with WBC levels. In contrast, these relationships were largely attenuated in the GDM group. Although *SHLP3* and *SHLP5* showed weak positive trends and *SHLP2* and *SHLP6* demonstrated moderate positive tendencies, none of these associations reached statistical significance.

Collectively, these exploratory within-group analyses indicate that placental *SHLP* transcript levels are closely associated with maternal metabolic status, particularly insulin resistance and glycemic control, as well as neonatal birth weight.

## Discussion

This study provides the first descriptive analysis of placental *SHLP1–6* expressions in pregnancies complicated by GDM. Our findings demonstrate a consistent and marked reduction in placental *SHLP* transcript levels in GDM, which is further supported by decreased circulating and placental levels of Humanin and SHLP2. These data extend current knowledge on mitochondrial genome-encoded peptides in diabetic pregnancy and identify dysregulated SHLP expression as a molecular feature associated with GDM. Collectively, the placenta emerges as a metabolically responsive tissue in which mitochondrial peptide signaling is closely linked to the altered maternal metabolic environment characteristic of GDM.

Mitochondrial function is increasingly recognized as an important determinant of placental physiology and pregnancy outcomes (1, 5, 10). Mitochondria are essential for placental function, governing energy production, redox balance, and apoptotic signaling in trophoblast cells (24). Alterations in placental mitochondrial structure and function have been reported in several pregnancy-related complications, including preeclampsia, intrauterine growth restriction, and preterm birth, conditions in which metabolic stress and oxidative imbalance are prominent features (24–26). In the context of GDM, placental tissues have been shown to exhibit metabolic stress accompanied by altered redox homeostasis, including increased oxidative burden and impaired antioxidant responses (27, 28). Structural abnormalities of placental mitochondria, such as swelling and disrupted cristae architecture, have also been described in GDM pregnancies, consistent with mitochondrial stress or injury (29). Within this context, the observed reduction in SHLP transcripts and peptides is consistent with mitochondrial perturbations in the diabetic intrauterine environment. Because SHLPs are encoded within mtDNA, reduced *SHLP* transcripts in GDM placentas may also partly reflect broader mitochondrial alterations (e.g. mitochondrial content/mtDNA-associated transcription) (30). Our expanded analyses suggest that *SHLP* downregulation is not merely a consequence of global mitochondrial transcriptional decline. Although mitochondrial transcripts (*12S rRNA, 16S rRNA*, and *CO1*) exhibited mild, non-significant reductions in GDM placentas, the magnitude of SHLP downregulation was substantially greater and remained significant after normalization to mitochondrial markers. These findings indicate that the observed decrease in SHLP expression likely reflects a more specific regulatory mechanism rather than a generalized reduction in mitochondrial RNA abundance. This distinction is critical, as it supports the hypothesis that mitochondrial peptide signaling is selectively altered in GDM rather than passively reflecting mitochondrial content. Nevertheless, it should be acknowledged that some *SHLP*-targeting primer pairs amplify partially overlapping regions within the *16S rRNA* transcript. In particular, the *SHLP1* and *SHLP4* primer sets target overlapping regions; therefore, the corresponding qPCR signals may not represent completely independent transcript species. Accordingly, *SHLP1* and *SHLP4* results should be interpreted as changes in overlapping SHLP-encoding regions of the parental *16S rRNA* transcript rather than as evidence for distinct monocistronic *SHLP* transcripts.

Mitochondria have recently been recognized as contributors to cellular communication and metabolic regulation through the production of short peptides encoded within the mitochondrial genome, collectively referred to as MDPs (31). MDPs are now known to influence systemic metabolism, inflammatory pathways, cell survival, and insulin sensitivity (32–34). The SHLPs were discovered more recently as additional mitochondrial signals, and early studies suggested that they too play roles in modulating metabolism and cell fate (13, 31). However, data regarding MDP expression and regulation in pregnancy, particularly at the level of the placenta, remain limited. Pregnancy is characterized by profound metabolic adaptations, including physiological insulin resistance, and recent clinical observations indicate that circulating levels of certain MDPs, such as MOTS-c, vary across gestation and are associated with maternal insulin sensitivity (23). Previous clinical studies have reported that circulating levels of Humanin and MOTS-c are reduced in individuals with type 2 diabetes and are inversely associated with markers of glycemic control, including HbA1c and insulin resistance (23, 35, 36). These observations support the notion that altered MDP profiles accompany states of chronic hyperglycemia and metabolic stress. GDM shares key pathophysiological features with type 2 diabetes, particularly insulin resistance and low-grade inflammation (1, 29). Our findings extend these observations by demonstrating that both circulating and placental levels of Humanin and SHLP2 are reduced in GDM pregnancies. The concordance between transcript and peptide-level alterations strengthens the biological relevance of our findings and suggests that impaired MDP signaling may contribute to the metabolic dysregulation observed in GDM.

Previous experimental studies have highlighted SHLP2 and SHLP3 as functionally relevant members of the SHLP family, reporting effects related to cellular stress responses and metabolic pathways in *in vitro* and animal models (13, 31). In these settings, *SHLP2* and *SHLP3* have been associated with reduced apoptosis, modulation of oxidative stress, and changes in mitochondrial function, whereas *SHLP6* has been described to exert opposing, pro-apoptotic effects (13, 31). The differential expression patterns observed in the present study may therefore reflect distinct functional roles of individual SHLPs in placental adaptation to metabolic stress. In addition, the inverse relationship between SHLP levels and neonatal birth weight indicates a potential role for SHLPs in the regulation of fetal growth, especially under conditions of maternal metabolic imbalance. Similar associations between mitochondrial-derived peptides and fetal growth parameters have been reported previously (37), supporting a broader role for MDPs in fetal–placental metabolic crosstalk. In particular, *SHLP1–3* consistently demonstrated strong inverse correlations with key metabolic indices, including HOMA-IR, HbA1c, and neonatal birth weight, across both control and GDM groups. *SHLP4* showed a more variable pattern, with significant associations observed primarily in the GDM group. Notably, *SHLP5* also exhibited significant inverse correlations with HbA1c and birth weight in GDM pregnancies, despite showing weaker or non-significant associations with HOMA-IR. In contrast, *SHLP6* displayed more modest and less consistent relationships across the analyzed parameters.

By contrast, associations between placental *SHLP* expression and maternal WBC counts were modest, consistent with the view that leukocytosis in GDM reflects a low-grade, chronic inflammatory state rather than acute inflammation (38). Maternal WBC counts are commonly used as a surrogate marker of low-grade systemic inflammatory activity in metabolic disorders (39). Accordingly, the weak *SHLP*–WBC correlations observed suggest that placental *SHLP* expression is more closely related to metabolic than inflammatory alterations in GDM.

Taken together, the present findings provide new insights into the role of mitochondrial-derived peptides in GDM. The consistent reduction in *SHLP* transcripts and peptides, their strong associations with metabolic parameters, and their relative independence from global mitochondrial transcriptional changes collectively support a model in which mitochondrial peptide signaling is selectively impaired in GDM. Further studies incorporating longitudinal sampling, mechanistic experiments, and broader peptide profiling will be necessary to determine whether SHLPs have diagnostic or therapeutic potential in the management of gestational diabetes.

## Conclusion

This study demonstrates that placental SHLP transcript levels are markedly reduced in pregnancies complicated by GDM. This transcriptional downregulation is accompanied by decreased circulating and placental levels of Humanin and SHLP2, supporting the biological relevance of these findings at the peptide level. Moreover, *SHLP* expression was closely associated with key maternal metabolic parameters, including insulin resistance and glycemic control, as well as neonatal birth weight, highlighting a potential link between placental mitochondrial peptide signaling and the metabolic environment of GDM. Importantly, the observed reduction in SHLP expression persisted after normalization to mitochondrial transcripts, suggesting that these alterations are not solely attributable to global changes in mitochondrial RNA abundance but may reflect more specific regulatory mechanisms affecting mtDNA-encoded peptide expression. Despite these strengths, the study remains observational in nature and does not establish causality. Future studies incorporating longitudinal designs, broader peptide profiling, and functional approaches will be necessary to elucidate the mechanistic and potentially translational role of SHLPs in placental adaptation and their potential diagnostic or therapeutic relevance in GDM.

## Supplementary Material



## Data Availability

The datasets generated and/or analyzed during the current study are available from the corresponding author on reasonable request.
