# Supplementary material for: Altered placental expression of small humanin-like peptides in gestational diabetes mellitus
Source: Ups J Med Sci. 2026 Jul 3;131:10.48101/ujms.v131.14142. doi: 10.48101/ujms.v131.14142 (PMC13366712; doi:10.48101/ujms.v131.14142)
Supplement: Supplementary file 1 [file UJMS-131-14142-s1.pdf]

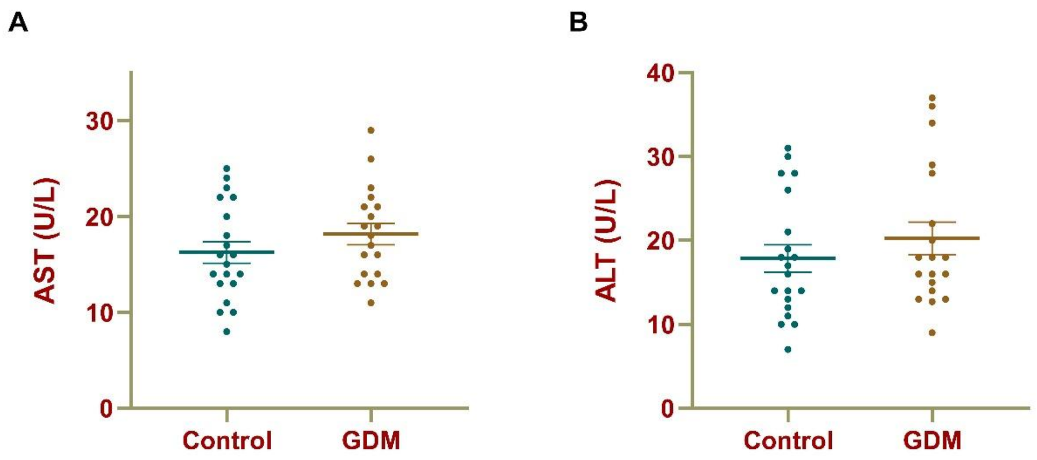

Supplementary Figure 1. Serum liver enzyme levels in control and GDM groups during the third trimester

Maternal serum levels of (A) aspartate aminotransferase (AST) and (B) alanine aminotransferase (ALT) were measured in fasting venous blood samples collected during the third trimester. Each data point represents an individual participant (control, n = 20; GDM, n = 19). Statistical comparisons between groups were performed using the Mann–Whitney U test. Data are expressed as mean ± SEM.

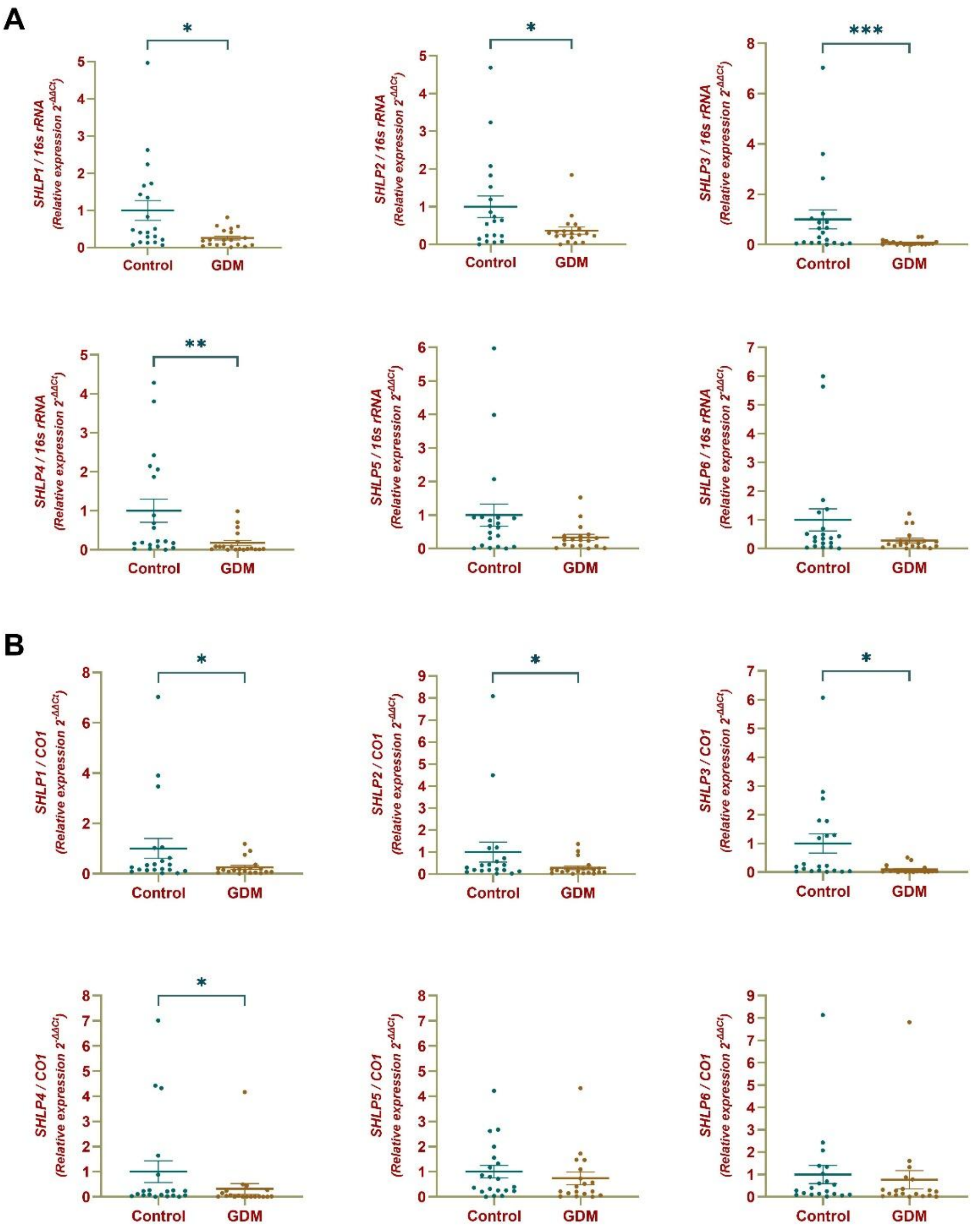

Supplementary Figure 2. Normalization of *SHLP* transcript levels to mitochondrial markers in control and GDM groups

Relative expression levels of *SHLP1–6* normalized to mitochondrial transcripts in placental tissues from control (n = 20) and GDM (n = 19) pregnancies. (A) *SHLP* expression normalized to *16S rRNA* and (B) *SHLP* expression normalized to *CO1*. Gene expression levels were calculated using the $2^{-\Delta\Delta Ct}$ method and are presented relative to the mean expression of the control group, which was set to 1. Each reaction was performed in triplicate. Data are presented as mean ± SEM. *P < 0.05, **P < 0.01, ***P < 0.001 vs control (Mann–Whitney U test).

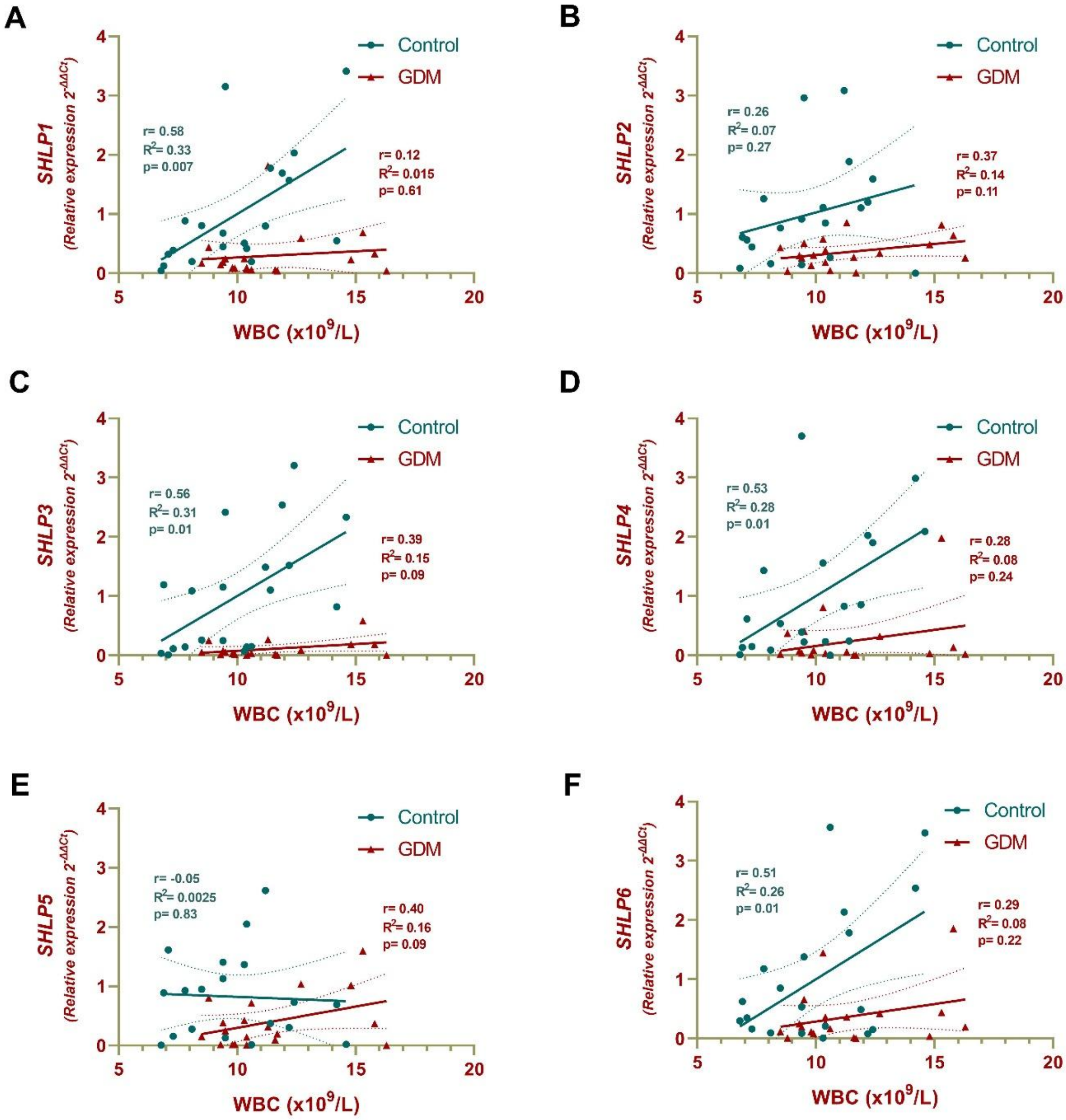

Supplementary Figure 3. Correlations between placental *SHLP1–6* transcript levels and maternal WBC counts during the third trimester

Correlation analysis between placental mRNA expression of A) *SHLP1*, (B) *SHLP2*, (C) *SHLP3*, (D) *SHLP4*, (E) *SHLP5*, and (F) *SHLP6* and maternal white blood cell (WBC) counts measured during the third trimester in control and GDM pregnancies. *SHLP* transcript levels were quantified by qPCR, normalized to the geometric mean of *ACTB* and *GAPDH*, and expressed as relative values calculated using the $2^{-\Delta\Delta Ct}$ method, with the control group used as the calibrator. Each data point represents an individual placenta (control, n = 20; GDM, n = 19). Pearson correlation analysis was performed to assess associations between variables within each group. Linear regression lines are shown for visualization purposes. The correlation coefficient (r), coefficient of determination (R^2), and p-values were calculated using Pearson's correlation test. Dotted lines represent 95% confidence intervals.

Table 1. Patient clinical characteristics

	**Control (n=20)**	**GDM (n=19)**
**Age (y)**	30.85 ± 4.78	31.37 ± 4.21
**1st Trimester BMI (kg/m^2)**	25.81 ± 2.03	26.53 ± 2.09
**2nd Trimester BMI**	26.35 ± 2.20	27.28 ± 1.45
**3rd Trimester BMI**	27.02 ± 1.68	28.27 ± 1.21
**1st Trimester White Blood Cell (x10^9/L)**	9.13 ± 2.02	8.94 ± 1.71
**2nd Trimester White Blood Cell**	9.57 ± 1.65	9.62 ± 1.84
**3rd Trimester White Blood Cell**	10.00 ± 2.34	11.39 ± 2.44
**1st Trimester HbA1c (%)**	4.68 ± 0.43	4.94 ± 0.38
**2nd Trimester HbA1c**	4.94 ± 0.70	5.57 ± 0.48
**3rd Trimester HbA1c**	5.14 ± 0.49	**6.55 ± 0.46***
**3rd Trimester Insulin (μIU/mL)**	12.98 ± 0.64	**17.41 ± 0.89***
**Intrapartum Amniotic Fluid Index (cm)**	10.65 ± 2.64	11.68 ± 4.29
**Gestational Age (weeks)**	37.80 ± 1.32	37.53 ± 0.96
**Birth Weight (g)**	3070 ± 339.6	3188 ± 371.9
**Apgar (1 min)**	8.10 ± 0.85	7.73 ± 0.87
**Apgar (5 min)**	9.00 ± 0.79	8.84 ± 0.76
**75 g OGTT (~ 28. weeks of gestation)**		
**Fasting (mg/dL)**	80.65 ± 7.94	**102.7 ± 10.85***
**1 h**	124.3 ± 10.54	**191.7 ± 10.72***
**2 h**	103.5 ± 7.45	**169.7 ± 13.45***

Data are presented as mean ± SD. Significant differences were found *P < 0.05 compared with control. Control, normal pregnant women; GDM, gestational diabetes mellitus; BMI, body mass index; HbA1c, glycated hemoglobin; OGTT, oral glucose tolerance test; Apgar (appearance, pulse grimace response, activity, respiration) newborn's status after birth. Statistical analysis was performed using unpaired Student's t-test.

Supplementary Table 1. *Forward and reverse primer sequences for mitochondrial SHLP transcripts (SHLP1–6)*

Gene	***Forward (5'-3')***	***Reverse (5'-3')***
SHLP1	*AAATCTTACCCCGCCTGTTT*	*ACCTTTGCACGGTTAGGGTA*
SHLP2	*TGGTGATAGCTGGTTGTCCA*	*AGGCTTATGCGGAGGAGAAT*
SHLP3	*ATTGAAACCTGGCGCAATAG*	*TGGACAACCAGCTATCACCA*
SHLP4	*GCATAAGCCTGCGTCAGATT*	*CTACCTTTGCACGGTTAGGG*
SHLP5	*CCTAACAAACCCACAGGTCCT*	*GGATTGCGCTGTTATCCCTA*
SHLP6	*ACCTCGATGTTGGATCAGGA*	*CCTGGATTACTCCGGTCTGA*
12S rRNA	*TAGAGGAGCCTGTTCTGTAATCGAT*	*CGACCCTTAAGTTTCATAAGGGCTA*
16S rRNA	*CCTCCCTGTACGAAAGGACAAGAG*	*CTGTTCTTGGGTGGGTGTGGGT*
CO1	*GTTGTAGCCCACTTCCAC*	*CATCGGGGTAGTCCGAGTAA*
ACTB	*AACTGGGACGACATGGAGAA*	*GAAGGTCTCAAACATGATCTGG*
GAPDH	*GGAGCGAGATCCCTCCAAAAT*	*GGCTGTTGTCATACTTCTCATGG*
